# Supplementary material for: Relationship between Lifestyle Determinants and Perceived Mental and Physical Health in Italian Nursery and Primary School Teachers after the COVID-19 Lockdown
Source: J Funct Morphol Kinesiol. 2024 Feb 17;9(1):33. doi: 10.3390/jfmk9010033 (PMC10885123; doi:10.3390/jfmk9010033)
Supplement: Supplementary file 1 [file jfmk-09-00033-s001.zip › Table S1.pdf]

Table S1. Relationship between weight status and physical activity levels, adherence to MD and physical and mental health.

	**Normal-weight**			**Overweight**			**Obese**		
	**TS**	**NS**	**PS**	**TS**	**NS**	**PS**	**TS**	**NS**	**PS**
**Physical activity levels**									
**Inactive**	327±211	308±237,5	350±181	426±154	465±143,3	398±166	272±123	210±84,9	395± -
**Sufficiently Active**	1641±541	1609±535,7	1668±552	1342±459	1395±504,9	1278±417	1604±484	1380±505	1717±477
**Active or highly active**	5094±2773	5410±3373	4936±2435	3935±1515	3865±1558	4011±1531	5898±3044	7613±1601.6	5040±3414
**MD[a] Adherence (score)**									
**MD[a]**	8.90±1.97	9.14±2.09	8.73±1.88	8.82±1.82	9.03±1.55	8.61±2.08	8.56±1.38	8.14±1.68	8.82±1.17
**SF-12 (score)**									
**PCS[b]**	50.12±7.33	49.39±7.59*	50.65±7.13	49.11±8.04	47.51±8.21	50.76±7.64	46.36±6.89	42.72±5.43*	48.68±6.91
**MCS[c]**	51.42±8.17	51.70±8.03	51.23±8.29	51.30±8.42	52.23±6.75	50.34±9.90	55.03±4.38	56.20±5.32	54.28±3.75

a, Mediterranean Diet; b, Physical Component Summary; c, Mental Component Summary; $*p < 0.05$; $**p < 0.01$ $***p<0.001$
