# Supplementary material for: Relationship between Lifestyle Determinants and Perceived Mental and Physical Health in Italian Nursery and Primary School Teachers after the COVID-19 Lockdown
Source: J Funct Morphol Kinesiol. 2024 Feb 17;9(1):33. doi: 10.3390/jfmk9010033 (PMC10885123; doi:10.3390/jfmk9010033)
Supplement: Supplementary file 1 [file jfmk-09-00033-s001.zip › Table S2.pdf]

Table S2. Relationship between educational levels and physical activity levels, adherence to MD and physical and mental health.

	Secondary School			Graduation Degree		
	**TS**	**NS**	**PS**	**TS**	**NS**	**PS**
**Physical activity levels**						
**Inactive**	384±190	363±217	408±159	301±198	287±229	316±175
**Sufficiently Active**	1602±545	1508±564	1700±520	1558±521	1579±496	1542±548
**Active or highly active**	5248±2808	5437±3020	5118±2686	4534±2364	4592±2991	4505±2024
**MD[a] Adherence (score)**						
**MD[a]**	8.81±1.84	9.03±1.91	8.61±1.77	8.91±1.97	9.08±2.01	8.80±1.95
**SF-12 (score)**						
**PCS[b]**	49.76±7.49	48.41±7.64	50.96±7.20	49.54±7.53	48.62±7.99	50.15±7.19
**MCS[c]**	52.66±7.27*	52.25±7.15	53.03±7.41**	50.62±8.67*	51.93±8.19	49.76±8.92**

a, Mediterranean Diet; b, Physical Component Summary; c, Mental Component Summary; *p < 0.05; **p < 0.01 ***p<0.001
